# Unsteady Convection Flow and Heat Transfer over a Vertical Stretching Surface

**DOI:** 10.1371/journal.pone.0107229

**Published:** 2014-09-29

**Authors:** Wenli Cai, Ning Su, Xiangdong Liu

**Affiliations:** 1 Department of Mathematical Sciences, Tsinghua University, Beijing, China; 2 Department of Financial Engineering, Donlinks School of Economics and Management, University of Science and Technology Beijing, Beijing, China; China University of Mining and Technology, China

## Abstract

This paper investigates the effect of thermal radiation on unsteady convection flow and heat transfer over a vertical permeable stretching surface in porous medium, where the effects of temperature dependent viscosity and thermal conductivity are also considered. By using a similarity transformation, the governing time-dependent boundary layer equations for momentum and thermal energy are first transformed into coupled, non-linear ordinary differential equations with variable coefficients. Numerical solutions to these equations subject to appropriate boundary conditions are obtained by the numerical shooting technique with fourth-fifth order Runge-Kutta scheme. Numerical results show that as viscosity variation parameter increases both the absolute value of the surface friction coefficient and the absolute value of the surface temperature gradient increase whereas the temperature decreases slightly. With the increase of viscosity variation parameter, the velocity decreases near the sheet surface but increases far away from the surface of the sheet in the boundary layer. The increase in permeability parameter leads to the decrease in both the temperature and the absolute value of the surface friction coefficient, and the increase in both the velocity and the absolute value of the surface temperature gradient.

## Introduction

Convection and heat transfer in porous medium appear in many disciplines, such as thermal and insulation engineering, geophysics and chemistry. In the past few decades, the study in this area has attracted extensive attention of many researchers. Raptis [1] discussed the influence of the radiation on free convection flow through bounded porous medium. Afify [2] analyzed non-Darcy free convection flow through a non-isothermal impermeable vertical plate embedded in a thermally stratified porous medium. Hayat et al. [3]. considered magnetohydrodynamic stagnation-point flow pass a stretching vertical plate with thermal radiation in a porous medium and analyzed the existence of solution by using homotopy analysis method. Convective heat transfer of incompressible viscous fluid over a porous wedge embedded in porous medium was investigated by Anbuchezhian et al. [4]. Mukhopadhyay and Layek [5] discussed the effect of variable viscosity on the boundary layer flow and heat transfer of a fluid through a porous medium. Moreover, Some researchers considered the influence of radiation on heat and mass transfer of steady MHD flow (see [6]–[11]).

The above-mentioned studies [1]–[11] concerned about steady fluid flow. However, the flow and heat transfer is at unsteady conditions in many practical situations, for example, because of a sudden stretching of the plate or due to temperature change of the plate. When the surface is extended suddenly with a certain speed, the flow in the viscous boundary layer near the plate is slowly developed and evolved into a fully developed steady flow after a period of time. So it is necessary to consider physical quantity related to time in mathematical modeling. Ishak et al. [12] studied unsteady laminar boundary layer flow over stretching permeable surface. Further, Tsai et al. [13] and Abd El-Aziz [14] analyzed the impact of radiation on the unsteady flow and heat transfer over stretching surface. In fact, the combined effects of radiation and magnetic field on unsteady viscous incompressible fluid flow have been considered by some scholars (see [15]–[17]).

In addition, when the unsteady stretching surface is located in porous medium, the impact of different factors on the heat transfer is discussed in some recent works. For instance, in the presence of a magnetic field, the viscous incompressible conductive fluid flow along a semi-infinite vertically porous moving plate is researched by Kim [18]. Israel-Cookey et al. [19] analyzed the influence of radiation and viscous dissipation on unsteady MHD free convection flow. For unsteady MHD flow pass a porous vertical plate immersed in porous medium, Samad and Mansur-Rahman [20] considered the combined impact of radiation and magnetic field on MHD free convection flow. Elbashbeshy et al. [21] studied unsteady convective flow over porous stretching surface in the porous medium in the presence of heat source or sick. On this basis, Pal and Hiremath [22] discussed the impact of the dissipation on the heat transfer of fluid flow.

Similar to steady condition, the process of unsteady fluid flow not only includes heat transfer but also mass transfer. When there exists the heat source or sink, Ibrahim et al. [23] analyzed the combined impact of chemical reaction and radiation on heat and mass transfer of MHD flow. Some researchers considered the influence of magnetic field, the radiation, chemical reaction and their combined effects on heat and mass transfer (see [24]–[27]).

According to physical properties of most of realistic fluids, the viscosity and the thermal conductivity are usually related to the temperature and may vary dramatically with temperature. Thus, it would be more reasonable to take into account the effects of temperature dependent viscosity and thermal conductivity in mathematical modeling so as to accurately predict the flow behaviour. In view of this, Seddeek [28] studied the effects of radiation and variable viscosity on the magnetic fluid flow. Solving controlling partial differential equations by using the finite difference method, he further analyzed heat transfer characteristics of fluid flow in the boundary layer. Mukhopadhyay [29] considered the impact of the radiation on unsteady mixed convection boundary layer flow, the effects of the various physical parameters on the velocity and temperature are investigated by using the numerical analysis. By considering assisting and opposing buoyant flow situations, Vajravelu et al. [30] discussed the influence of variable thermal conductivity and radiation on unsteady convective and heat transfer.

To the best of the author's knowledge, no attempt has been made to analyze the combined effects of both temperature dependent viscosity and thermal conductivity on unsteady convection and heat transfer over a vertical permeable stretching surface in porous medium in the presence of radiation. Hence, the aim of the present investigation is design a suitable physical model to describe unsteady two-dimensional incompressible viscous fluid flow past a vertical permeable sheet in a porous medium and solve such an issue numerically using Runge-Kutta fourth-fifth order method with secant shooting technique, which is important from both theoretical and practical point of view because of its wide application to polymer technology and metallurgy. And finally the impact of various physical parameters (such as viscosity variation parameter, the variable thermal conductivity parameter, unsteady parameter, permeability parameter, the suction or injection parameter, convection parameter, thermal radiation parameter, Prandtl number) on the velocity and temperature profiles is displayed in the form of tables and graphs. It is hoped that the results obtained from the present investigation will provide useful information for application and also serve as an effective complement to the previous studies.

## The Construction and Analysis of the Model

**Figure 1:**
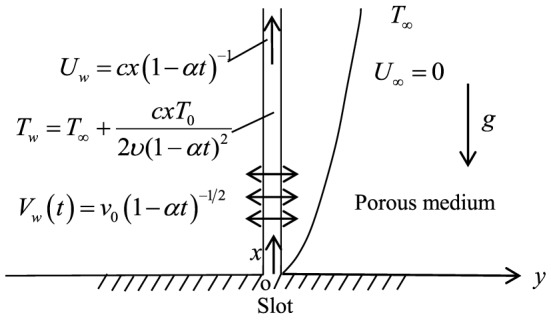
A schematic representation of mathematical model.

Consider convection and heat transfer of the unsteady two-dimensional incompressible viscous fluid flow along a vertical permeable sheet in a porous medium. The origin of the Cartesian coordinates 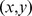 is the slot position, which is shown in Fig.1.

The 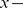 and 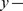 axis are taken in the direction along the sheet and perpendicular to it, respectively. Assuming flow region is restricted in the plane 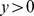. It is assumed that the magnetic Reynolds number of the flow is extremely small so that the induced magnetic field is negligible, which is a valid assumption on a laboratory scale(see Muhaimin et al. [31]). Simultaneously, suppose that no external electric field and magnetic field are applied, Edge effect and hall effect are also negligible. All dependent variables will be independent of the y-direction (Gorla and Sidawi [32]). The sheet moves with the velocity 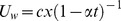 in its own plane, where 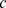 and 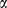 are constants with dimension reciprocal time and satisfying that 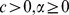 and 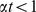. The flow is generated by the movement of the sheet; surface temperature of the sheet is



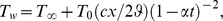
where 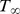 is the temperature of the fluid outside the boundary layer; 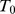 is constant; 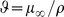 is the kinematic viscosity of the ambient fluid, here 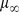 is the dynamic viscosity of ambient fluid and 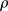 is the density of the fluid. 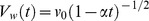 is the velocity of suction if 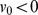 and injection if 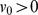. These particular forms of both 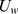 and 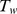 are chosen in order to obtain self-similar solutions of present problem. 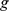 is the gravitational acceleration. The ambient fluid is stationary, that is 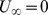. In addition, the temperature-dependent dynamic viscosity 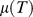 is assumed to linearly vary with the temperature in the form



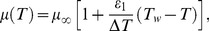
(1)where 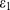 is a small viscosity variation parameter; 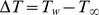; 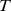 is the temperature of the fluid inside the thermal boundary layer. Also, it is assumed that the temperature-dependent thermal conductivity 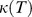 vary as a linear function of temperature given by the following (Vajravelu et al. [30])



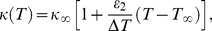
(2)where 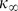 is thermal conductivity of the ambient fluid; 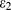 is a small variable thermal conductivity parameter; 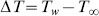.

Because of the behavior of the boundary layer, the temperature gradient along 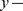 direction is much larger than that along 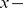 direction, therefore this paper only consider the velocity component of the thermal buoyancy which is vertical to the surface of sheet. Under Boussinesq's approximation and previous assumptions, the conservation equations governing this convective flow and heat transfer in boundary layer are



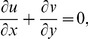
(3)




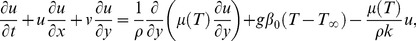
(4)




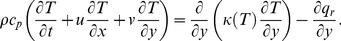
(5)


The appropriate boundary conditions to this physical problem are



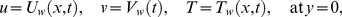
(6)




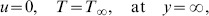
(7)where 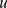 and 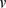 are the corresponding velocity components in 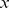 and 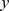 directions. 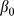 is the coefficient of thermal expansion. 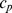 is the specific heat at constant pressure. 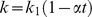 is the permeability of the porous medium. 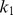 is the initial permeability. 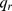 is the radiative heat flux. The second term on RHS of the momentum equation (4) denotes the thermal buoyancy effect. Also the first and second terms on the RHS of energy equation (5) represent heat conduction and the effect of thermal radiation, respectively.

Using Rosseland approximation for radiative heat flux term (Raptis [1]), this paper takes



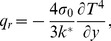
(8)where 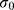 and 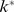 are the Stefan-Boltzman constant and the mean absorption coefficient, respectively. As described in the literature of Raptis ([1]), suppose that the temperature differences within the flow are sufficiently small, so 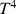 may be expressed as a linear function of temperature. This is accomplished by expanding 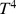 in a Taylor series about 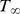 and neglecting higher-order terms, thus



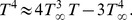
(9)


In view of (9), equation (8) is now reduced to



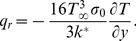
(10)


The similarity transformations for Eqs.(4)–(5) are as follows:



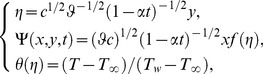
(11)where the stream function 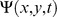 is defined by 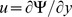 and 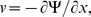 thus, the continuity equation (3) is satisfied automatically. By calculating, it is easy to obtain 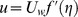. In the above and behind equations, 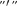 denotes the differentiation with respect to 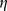 only. Here 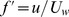 and 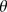 are the dimensionless velocity and temperature. Substituting (10)–(11) into (4)–(7), a set of ordinary differential equations with variable coefficients is given by



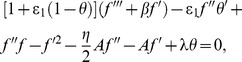
(12)




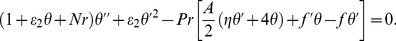
(13)


In the above equations, variable physical parameters are defined as



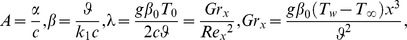





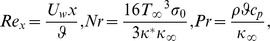
where 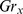 is the local Grashof number, 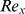 is the local Reynolds number, 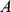 is unsteady parameter, 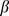 is the permeability parameter, 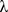 represents the convection parameter, 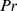 is the Prandtl number, 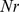 is thermal radiation parameter. All the symbols are defined in the Nomenclature.

The corresponding boundary conditions (6)–(7) for the velocity and temperature fields are transformed to



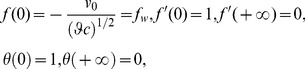
(14)where 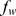 is suction or injection parameter which is used to control the strength and direction of normal flow at the boundary (Vajravelu et al. [30]).

Two physical quantities of interest in this problem are the surface friction coefficient 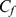 and the local Nusselt number 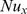, which are used to describe the characteristics of surface shear stress and the rate of heat transfer at the surface, respectively. The definitions are as follows



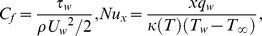
where 
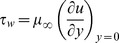
 and 
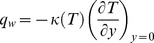
 are the shear stress along the surface and heat transfer from the sheet, respectively. Here 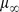 is viscosity coefficient of the ambient fluid and 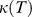 is the thermal conductivity of the fluid. The results of the calculation can be expressed as



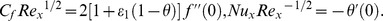



## Numerical Procedure

The boundary value problem of higher-order ordinary differential equations (12)–(14) will be transformed to the following initial value problem of first-order ordinary differential equations and will be solved numerically by using an efficient Runge-Kutta fourth-fifth order method with secant shooting technique under Matlab. Now new variables are defined by the equations



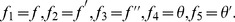
(15)


Thus, the above two coupled higher order differential equations (12)–(13) may be reduced to five equivalent first-order differential equations as follows



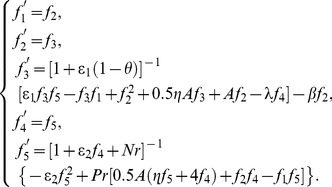
(16)


The boundary conditions (14) are converted to the following initial value conditions



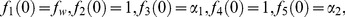
(17)where 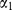 and 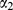 are the suitable initial guess values for 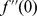 and 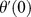, respectively. After choosing 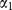 and 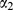, the initial value problems (16)–(17) are solved repeatedly by using Runge-Kutta fourth-fifth order numerical method with secant shooting technique until the boundary conditions 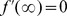 and 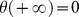 are satisfied. In the process of numerical solving (Ibrahim et al. [33]), the values of 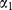 and 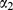 are improved and the position of the edge of the boundary layer 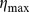 depending on nine unknown parameters had to be regulated to reach the accuracy. The step size of 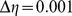 satisfies the convergence to the fifth decimal precision in almost all cases, which is sufficient for convergence of numerical solution.

In the next section, by using the above method, the detailed results of numerical simulation will be given to show the impact of various physical parameters on the absolute value of surface friction coefficient, the absolute value of the surface temperature gradient, the velocity and temperature profiles, respectively.

## Results and Discussion

In order to validate the method of this paper, ignoring the effects of 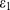 and 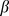, the model in this paper is the same as that in [30]. Vajravelu et al. [30] used a second order finite difference scheme known as the Keller-Box method to solve nonlinear ordinary differential equations subject to appropriate boundary conditions. Unlike their approach, this paper transforms the boundary value problem of higher-order ordinary differential equations to initial value problem of first-order ordinary differential equations and then uses Runge-Kutta fourth-fifth order numerical method with secant shooting technique to solve these equations numerically. The numerical simulation is employed to investigate the influence of critical parameters on both the absolute values of the surface friction coefficient 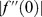 and the absolute values of the surface temperature gradient 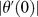, and the existing results are in excellent agreement. These favorable comparisons give confidence in the numerical method employed and the numerical results to be presented subsequently. Moreover, this paper analyzes the impact of 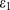 and 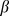 on 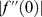 and 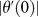. The obtained results are shown in Table 1.

**Table 1:** The effects of various parameters on 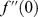 and 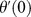.

	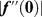	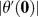
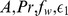	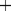	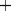
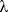	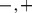	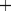
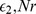	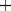	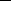
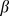	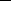	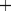

Note: 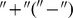 denotes as the physical parameters increase, 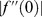 or 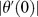 increases (decreases); 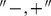 denotes that 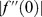 first decreases and then increases with the increase of 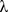.

More detailed results are shown in Tables 2–9, which reflects the effect of each parameter on both 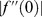 and 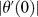. And also a representative set of numerical results is shown graphically in Figs.2–9 in order to illustrate the effects of various physical parameters on the velocity and temperature profiles.

**Figure 2:**
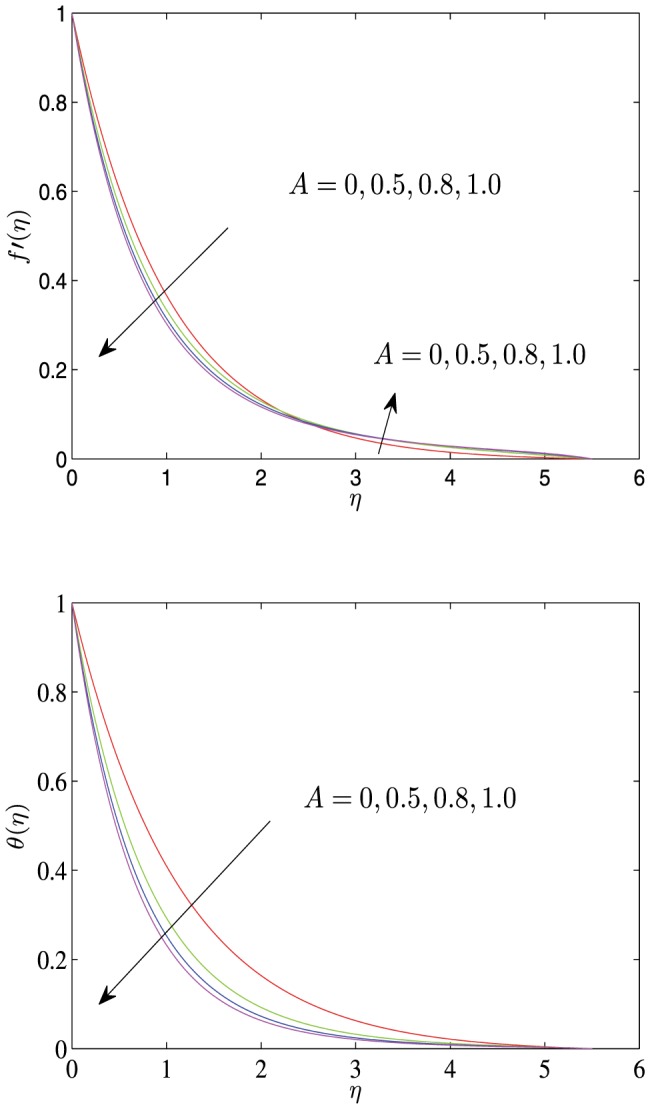
Horizontal velocity profiles 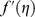 and temperature profiles 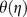 vs. 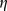 for different values of 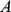 with 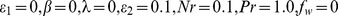.

**Figure 3:**
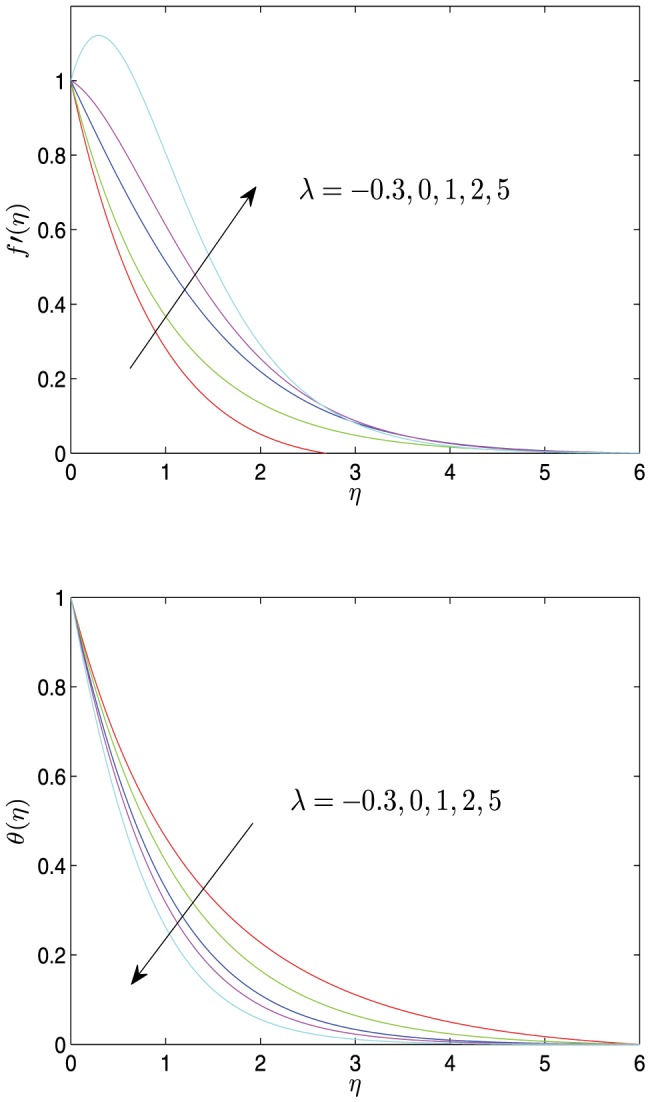
Horizontal velocity profiles 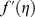 and temperature profiles 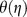 vs. 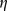 for different values of 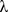 with 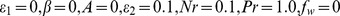.

**Figure 4:**
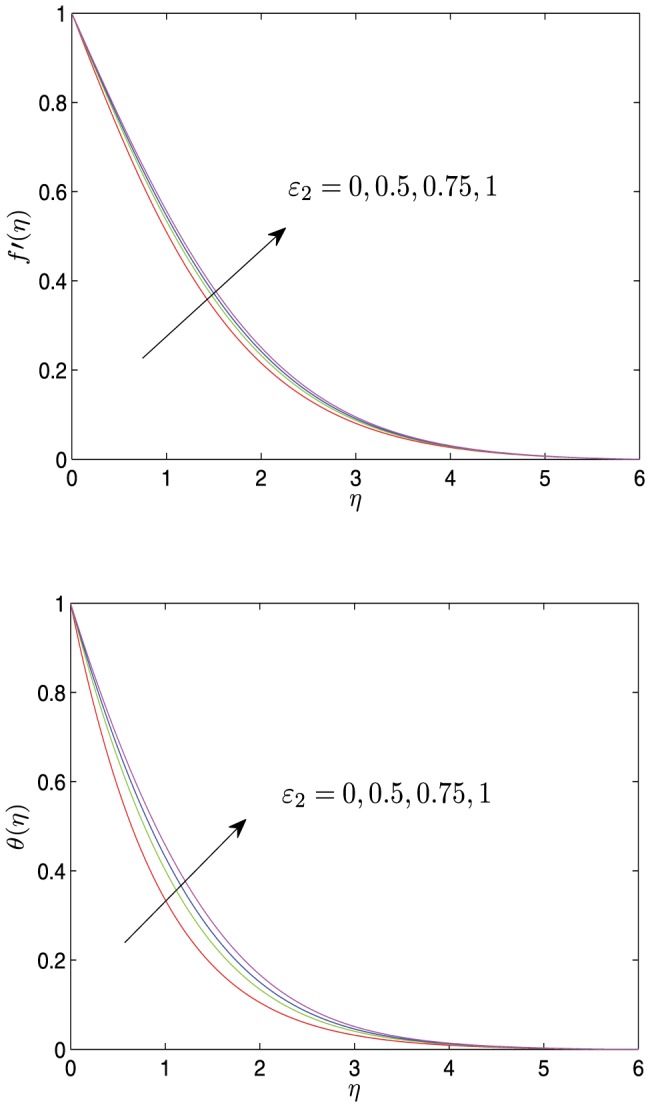
Horizontal velocity profiles 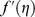 and temperature profiles 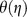 vs. 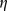 for different values of 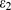 with 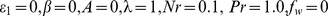.

**Figure 5:**
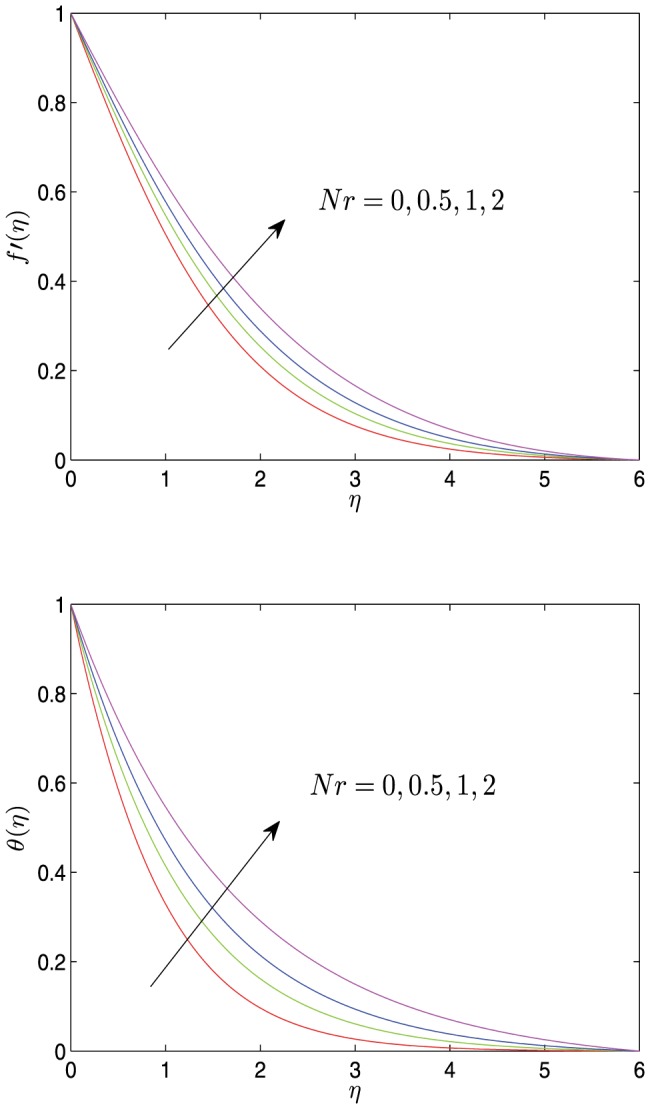
Horizontal velocity profiles 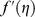 and temperature profiles 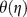 vs. 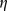 for different values of 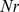 with 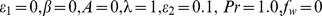.

**Figure 6:**
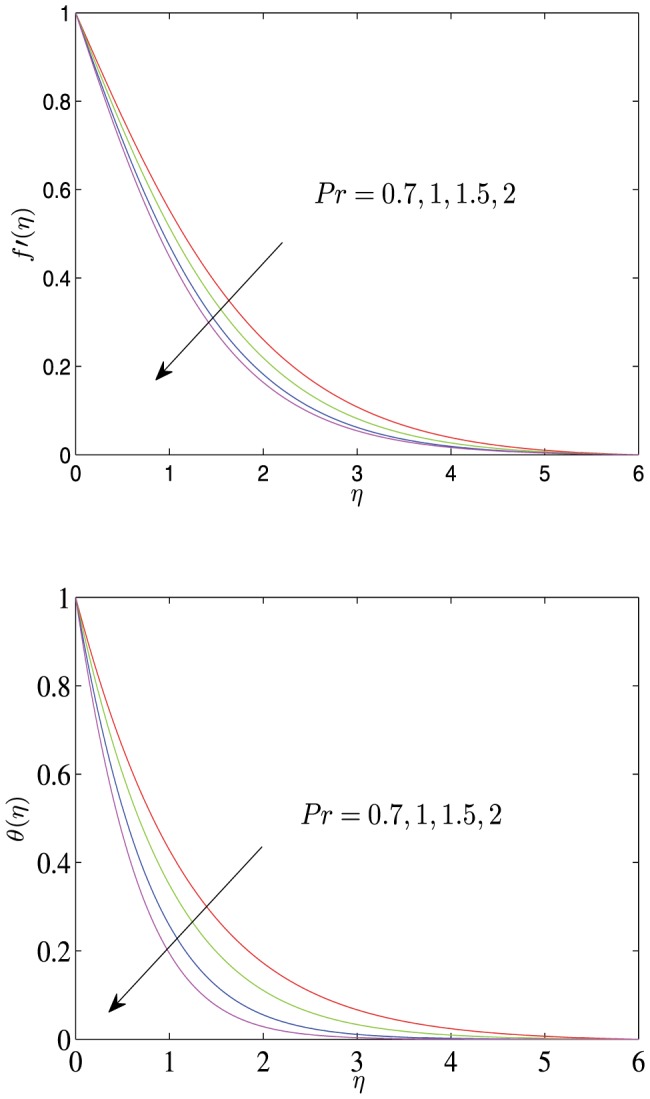
Horizontal velocity profiles 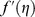 and temperature profiles 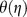 vs. 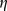 for different values of 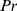 with 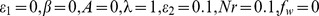.

**Figure 7:**
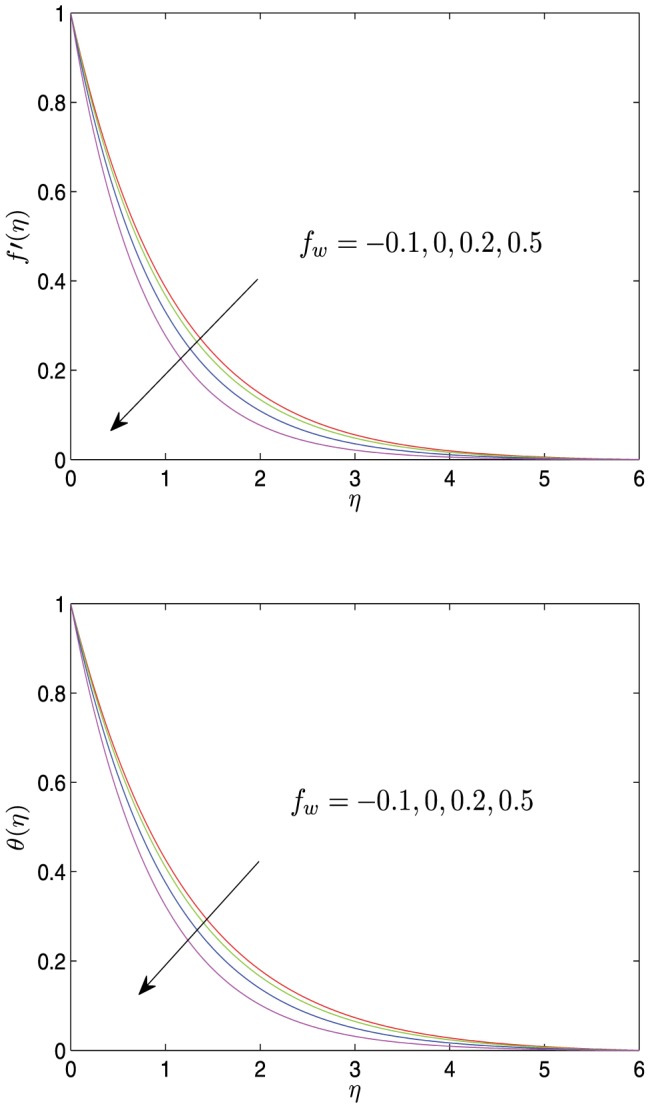
Horizontal velocity profiles 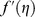 and temperature profiles 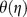 vs. 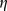 for different values of 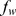 with 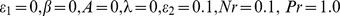.

**Figure 8:**
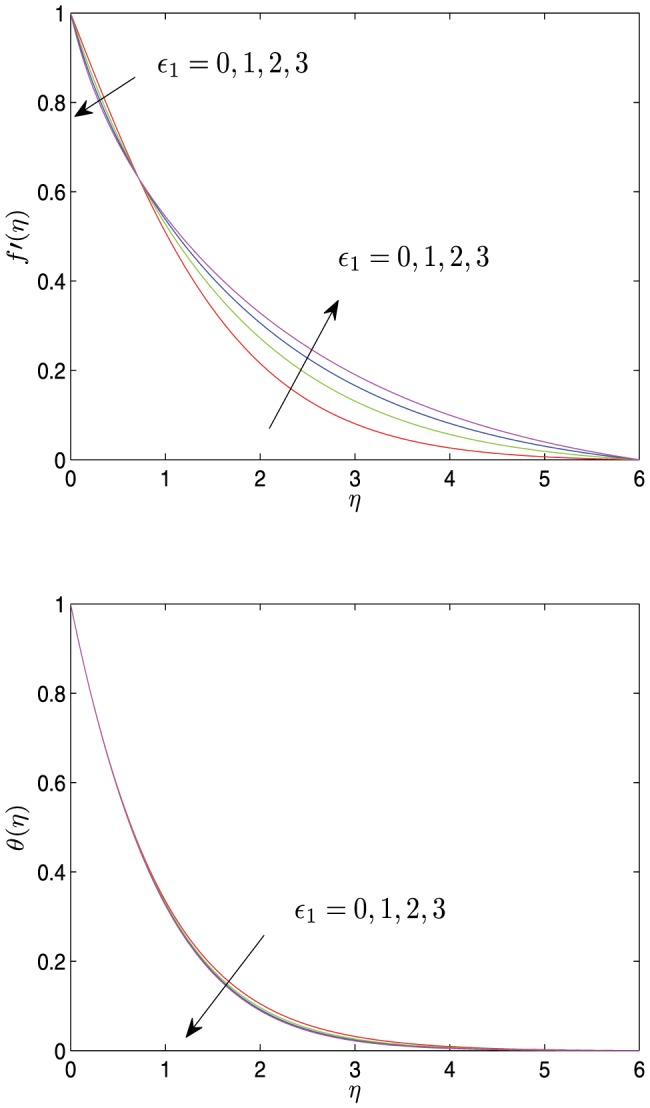
Horizontal velocity profiles 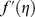 and temperature profiles 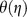 vs. 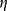 for different values of 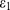 with 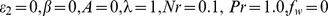.

**Figure 9:**
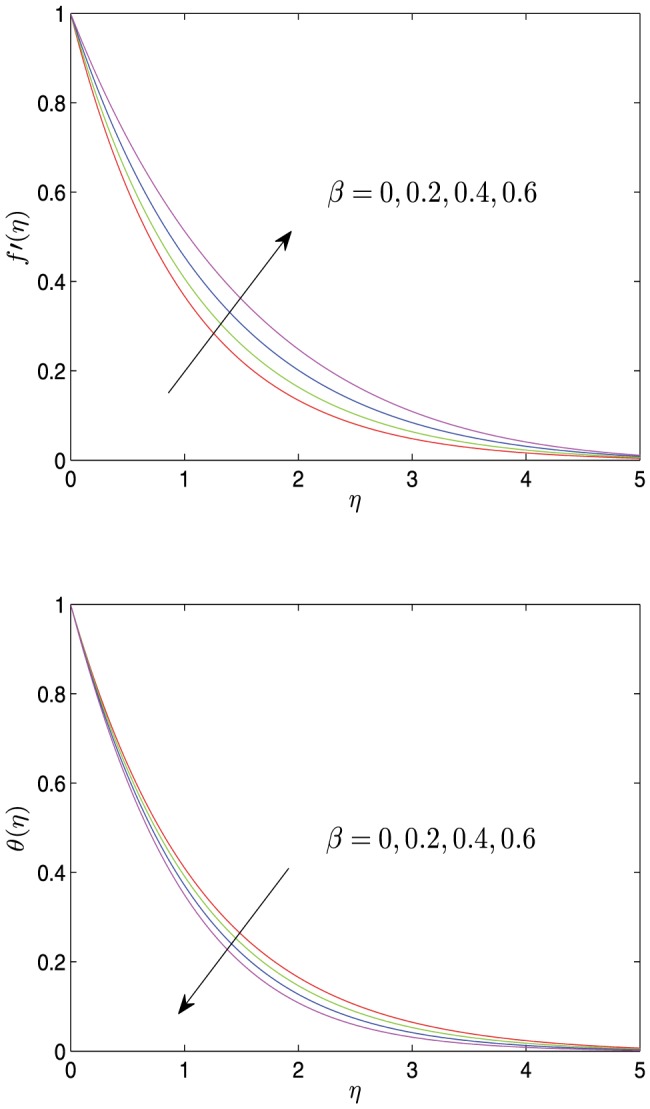
Horizontal velocity profiles 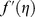 and temperature profiles 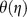 vs. 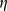 for different values of 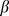 with 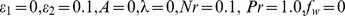.

**Table 2:** Comparison of values of 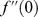 and 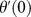 with previous results for different values of 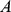 when 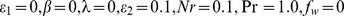.

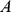	Vajravelu et al.([30])		Present results	
	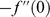	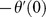	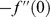	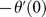
0	1.000489	0.883698	1.000484	0.883688
0.5	1.167325	1.241820	1.167324	1.241821
1.0	1.320522	1.507732	1.320559	1.507729
1.5	1.459660	1.731177	1.459990	1.731171

**Table 3:** Comparison of values of 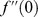 and 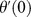 with previous results for different values of 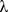 when 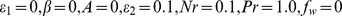.

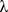	Vajravelu et al.([30])		Present results	
	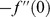	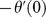	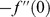	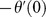
−0.3	1.173691	0.836590	1.175893	0.832248
0	1.000489	0.883698	1.000023	0.882719
0.5	0.756747	0.936146	0.756737	0.936147
1	0.538258	0.972561	0.537554	0.972733
2	0.139165	1.026678	0.139163	1.026678
5	−0.910504	1.133060	−0.910493	1.133058

**Table 4:** Comparison of values of 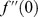 and 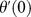 with previous results for different values of 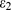 when 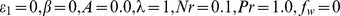.

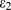	Vajravelu et al.([30])		Present results	
	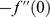	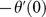	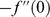	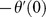
0.0	0.547387	1.032214	0.547379	1.032215
0.5	0.506138	0.802835	0.506131	0.802836
0.75	0.488913	0.730782	0.488906	0.730783
1.0	0.473401	0.674128	0.473393	0.674130

**Table 5:** Comparison of values of 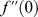 and 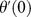 with previous results for different values of 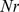 when 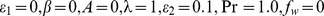.

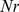	Vajravelu et al.([30])		Present results	
	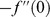	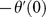	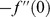	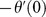
0.0	0.551459	1.018446	0.551453	1.018446
0.5	0.495188	0.834975	0.495172	0.834975
1.0	0.456107	0.723154	0.456078	0.723151
2.0	0.404490	0.589883	0.404434	0.589869

**Table 6:** Comparison of values of 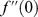 and 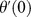 with previous results for different values of 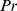 when 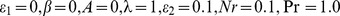.

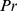	Vajravelu et al.([30])		Present results	
	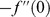	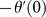	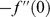	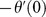
0.7	0.486030	0.801981	0.486012	0.801981
1	0.538258	0.972561	0.538251	0.972562
1.5	0.597645	1.212410	0.597642	1.212409
2.0	0.638053	1.417815	0.638051	1.417813

**Table 7:** Comparison of values of 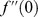 and 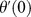 with previous results for different values of 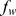 when 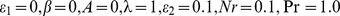.

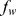	Vajravelu et al.([30])		Present results	
	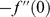	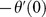	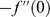	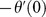
−0.1	0.951792	0.844385	0.951787	0.844375
0	1.000489	0.883698	1.000484	0.883688
0.2	1.124728	0.983985	1.105106	0.967828

**Table 8:** Comparison of values of 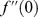 and 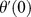 with previous results for different values of 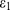 when 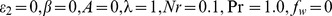.

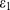	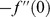	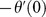
0.0	0.546852	1.032338
0.5	0.615513	1.033665
0.75	0.648432	1.034059
1.0	0.680574	1.034324

**Table 9:** Comparison of values of 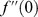 and 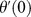 with previous results for different values of 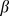 when 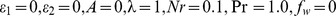.

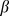	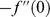	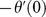
0.0	0.547379	1.032215
0.2	0.441123	1.052495
0.4	0.326559	1.074105
0.6	0.202389	1.097090

Fig.2 depicts the effects of various unsteady parameter 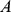 on velocity field and temperature field. As can be seen from these profiles, as the distance 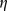 increases from 0, the velocity and the temperature monotonically decreasing tends to 0. As 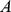 increases, the velocity 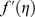 decreases near the sheet surface, but increases far away from the surface of the sheet in the boundary layer. The temperature and the temperature boundary layer thickness decrease with the increase of 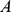, and simultaneously the temperature gradient increases. So the heat transfer rate of surface increases.

Fig.3 exhibits the velocity and the temperature profiles for different values of the convection parameter 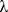. When 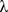 is very small, the velocity of the fluid decreases monotonically to 0. However, when the parameter 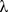 increases to a certain value, the velocity first monotonically increases to a peak and then monotonically decreasingly approaches to 0. The temperature decreases monotonically to 0 as the distance 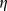 increases from 0. The increase in the values of 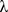 has the tendency to decrease the temperature and thermal boundary layer thickness but increase the temperature gradient.

Fig.4 represents the variation of both the velocity and temperature profiles in response to a change in the thermal conductivity parameter 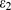. The graphs depict that both the velocity and the temperature decreasingly tends to 0 with the increase of values 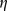. The velocity profiles increase slightly with an increase in 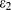. The increase of thermal conductivity parameter values leads to the increase of the temperature and the thermal boundary layer thickness, but simultaneously the decrease of the temperature gradient. In general, it can be seen from Fig.4 that the impact of the thermal conductivity parameter on the temperature field is more noticeable than that on the velocity field. Meanwhile, this phenomenon can be roughly observed from Eq.(13). Actually, 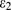 is the diffusion coefficient of the temperature and has a direct impact on the temperature. However, the impact of 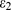 on the velocity is achieved through the coupling of the various terms, hence the effect may be weakened.

Figs.5–7 show the velocity and the temperature profiles with respect to the thermal radiation parameter 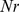, Prandtl number 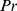 and the suction (or injection) parameter 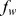. As shown in these graphs, both the velocity and the temperature decrease monotonically to 0 with the increase of 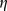. As 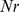 increases, both the velocity and the temperature increase, and both the velocity boundary layer and the temperature boundary layer become thicker, but the velocity gradient and the temperature gradient decrease. The varies of both 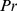 and 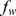 also affect the velocity, the velocity gradient, the velocity boundary layer thickness, the temperature, the temperature gradient and temperature boundary layer thickness, which is opposite to the effect of 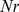 on the corresponding physical quantities.

Fig.8 reveals the impact of viscosity variation parameter 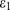 on the velocity and the temperature profiles. As can be seen from these profiles, as the distance 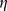 increases from 0, the velocity and the temperature monotonically decreasingly tend to 0. As 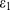 increases, the velocity 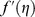 decreases near the sheet surface, but increases far away from the surface of the sheet in the boundary layer. The temperature profiles decrease slightly with an increase in 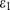 and simultaneously the temperature boundary layer thickness become thinner. From Fig.8, it is noticed that the effect of 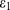 on the velocity is more pronounced than that on the temperature, which is different from thermal conductivity parameter. Similarly, this phenomenon can be roughly observed from Eq.(12). Furthermore, by comparing Fig.2 and Fig.8, it draws a conclusion that the effect of 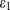 on the velocity and the temperature profiles is similar to that of 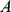 whereas is not as significant as that of 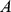.

Fig.9 demonstrates that the influence of the permeability parameter 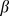 on the velocity and the temperature profiles. The results indicate that both the velocity and the temperature decreasingly tend to 0. As 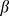 increases, the velocity increases and the velocity boundary layer thickens but the velocity gradient decreases. The impact of 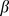 on the temperature, the temperature gradient and the thermal boundary layer thickness is opposite to that of 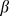 on the corresponding velocity.

## Conclusions

This paper investigates convection flow and heat transfer of an incompressible viscous fluid along a vertical permeable sheet through a porous medium. The resulting ordinary differential equations are solved numerically by using fourth-fifth order Runge-Kutta scheme with secant shooting method. The numerical results are presented for the major parameters including unsteady parameter 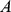, convection parameter 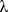, variable thermal conductivity parameter 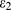, thermal radiation parameter 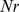, Prandtl number 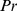, the suction or injection parameter 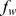, viscosity variation parameter 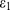 and permeability parameter 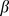. It finally analyzes the impact of the various parameters on flow and heat transfer characteristics.

Compared with the previous literature, this paper not only considers temperature-dependent viscosity and thermal conductivity, but also studies the effects of both the permeability of porous medium and radiation in mathematical modeling, which is helpful to accurately predict the flow behavior. Some novel numerical results are obtained as follows:

1. A different numerical method is used to investigate the impact of the physical parameters (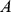, 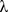, 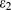, 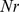, 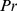 and 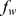) on the velocity and the temperature. The obtained results are similar to those in [30] where viscosity variation parameter and permeability parameter are not considered, which further verifies the accuracy of the obtained results.
2. Numerical results show that the velocity and temperature monotonously decrease to 0 with the increase of 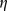 from the boundary, which means the velocity of the fluid far away from the boundary is close to the velocity of the ambient fluid which is assumed to be zero in this paper. This is quite consistent with the flow of actual fluid.
3. With the increase of viscosity variation parameter 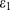, both the absolute value of the surface friction coefficient 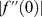 and the absolute value of the surface temperature gradient 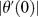 increase, where the increase of 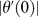 is very slow. Moreover, 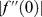 and 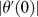 reach the minimum in the absence of 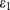. As 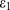 increases, the velocity decreases near the sheet surface, whereas increases far away from the surface of the sheet. During this process, the velocity gradient decreases but the velocity boundary layer thickness increases. The temperature profiles decrease slightly with an increase in 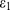 and simultaneously the temperature boundary layer becomes thinner. The influence of 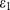 on the velocity is more pronounced than that on the temperature.
4. An increment in the permeability parameter 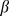 leads to the decrease of 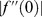 whereas the increase of 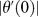. The increase in the values of 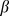 results in the increase of both the velocity and the velocity boundary layer thickness, which further leads to the decrease of the velocity gradient. But the effect of 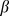 on the temperature, the temperature gradient and thermal boundary layer thickness is opposite to that of 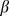 on the corresponding velocity.
